# Public Awareness, Attitudes, and First-Aid Measures on Epilepsy in Tehran

**Published:** 2019

**Authors:** Mohsen ABBASI KANGEVARI, Ali Asghar KOLAHI, Ahmad Reza FARSAR, Saeid KERMANIRANJBAR

**Affiliations:** 1 **Social Determinants of Health Research Center, Shahid Beheshti University of Medical Sciences, Tehran, Iran **; 2 **Student Research Committee, Shahid Beheshti University of Medical Sciences, Tehran, Iran**

**Keywords:** Behavior, Community, Health knowledge, Attitudes, Practice, Seizure, Superstition

## Abstract

**Objectives:**

People with epilepsy generally encounter misconceptions and negative attitudes on different aspects of the disease. They are also prone to physical injuries during seizures. Lack of awareness about first-aid measures results in taking inappropriate first-aid measures. We aimed to determine the public awareness, attitudes, and first-aid measures about epilepsy in Tehran.

**Materials & Methods:**

This population-based cross-sectional survey was conducted from Dec 2016 to May 2017 in Tehran, Iran. Random stratified cluster sampling was used. Data were collected through interviews using a questionnaire. The awareness section included general awareness, causes, symptoms, seizure triggers, first-aid measures, and recommended treatments. The Likert scale was used for the attitudes section which included 20 statements. The answers about first-aid measures were categorized as helpful, or harmful.

**Results:**

Overall, 833 adults participated in the survey. The level of total awareness score of 41 (4.9%) participants was very good, 194 (23.3%) good, 255 (30.6%) fair, 210 (25.2%) low, and 133(16.0%) very low. The mean (SD) score about general awareness was 4.6 (3.0), range=0 to 11; causes 5.8 (3.4), range=0 to 13; symptoms of seizures 7.0 (4.0), range=0 to 13; first-aid measures 7.5 (3.4), range=0 to 14. Among all participants, 260 (31.2%), named at least one superstitious cause for epilepsy. Attitudes were generally positive except for marriage and having kids. The level of first-aid measures score of 74(42.5) was very good, 79(45.4) good, and 21(12.1) low.

**Conclusion:**

The awareness of people of Tehran about epilepsy was insufficient, attitudes were generally positive but rather conservative, and first-aid measures at the last witnessed seizure were fairly helpful.

## Introduction

Epilepsy is still stigmatized in most parts of the world (1, 2). Public awareness, attitudes, and first-aid measures regarding epilepsy vary in different cultures. Nevertheless, people with epilepsy (PWE) generally encounter social stigma and public misconceptions about causes of epilepsy, its prognosis, treatment, and first-aid measures at time of seizures (3). 

Lack of public awareness about the nature of epilepsy is directly correlated with the presence of a stigma which results in negative attitudes towards PWE and impairs their quality of life more than the disease itself (2, 4). PWE may suffer from stigma and misconceptions in many aspects of life such as employment, education, and social relationships (5, 6). Proper awareness about epilepsy could prevent such misconceptions, stigma and experiencing worry and discomfort about PWE (7, 8).

PWE are prone to physical injuries subsequent to seizure (9). First-aid measures aim to protect PWE from getting harmed during a seizure. Lack of awareness and misconceptions about first-aid measures increase the chance of not taking helpful measures or taking harmful measures while witnessing a seizure (8). Moreover, to identify educational needs of the society, public awareness, and first-aid measures need to be assessed.

The objective of this population-based survey was to determine public awareness, attitudes, and first-aid measures towards epilepsy in Tehran, Iran. 

## Materials & Methods

This survey was approved by Ethics Committee of Shahid Beheshti University of Medical Sciences, Tehran, Iran on 16 Oct 2016 under the reference code IR.SBMU.REC.1395.505. Participants provided an informed consent before taking part in the survey and all data remained confidential. In addition, they were able to leave the interview at any stage.

**Setting and sampling**

This population-based descriptive survey was conducted from Dec 2016 to May 2017 in Tehran, Iran. There are nine million people living in the 22 municipality districts of Tehran. The sample size was set at 924 estimated based on the assumption that participants would gain 30% for first-aid measures, margin of error of 3% and 95% confidence interval. Random stratified cluster sampling was used. At first, we randomly selected 22 postal codes, a code for each of the 22 municipality districts of Tehran, using the Tehran post office database. We asked volunteer medical students to go to the addresses of the index postal code and invite residents to participate in the survey. Then, they would go on the left direction of their buildings to the front-door of their neighbors’ houses and invite all their neighbors to participate in the survey until the number of people who met the survey criteria reached 42 adults. 

**Participants**

Among 990 individuals who met the survey criteria, 883(89.2) accepted to participate in the survey. The inclusion criteria were being Iranian, living in Tehran, being able to communicate in Persian, being at least 18 yr old, and consenting to participate. Individuals with impaired ability to communicate were excluded.

**Variables** **and data collection**

Variables included socio-demographic characteristics of participants, their awareness about epilepsy, attitudes towards PWE, and first-aid measures at the last witnessed seizure. A structured questionnaire was used for data collection. The awareness section consisted of six categories including general awareness about epilepsy (11 items), causes (13 items), symptoms of seizures (13 items), seizure triggers (8 items), first-aid measures at time of seizures (14 items), and recommended treatments (7 items). Moreover, participants were asked how they evaluated their awareness, their sources of information, and whether they felt the need for further training in epilepsy.

The attitudes section included twenty statements about participants’ attitudes towards PWE. In response to these statements, participants chose an item from the five-point Likert scale. The first-aid measures section included questions about first-aid measures of participants, who had ever witnessed generalized seizures.

The item content was developed through a review of the literature, including published articles studying the same subject on the public (9-11), PWE (12,13), parents of children with epilepsy (14, 15), healthcare professionals (16, 17), teachers (18, 19), and students (20, 21); and open-ended interviews with eight experts in the field, including neurologists, public health experts, special educators, and rehabilitation counselors.

Content validity of the questionnaire was evaluated by a group of five neurologists, public health and health education experts. An item discrimination analysis was conducted for each scale to eliminate too difficult and too easy items. Factor analysis was performed for factor structure. Separate test-retest over a two-week period were conducted for the three scales of the questionnaire. Test-retest correlation for the awareness scale was 0.87; Kuder-Richardson-20 was used to prevent overestimation of internal consistency, coefficient was 0.83. Test-retest correlation for the attitudes scale was 0.89; coefficient alpha was 0.9. Test-retest correlation for the scale regarding first-aid measures was 0.93; coefficient alpha was 0.95. The pilot survey was conducted on fifty men and fifty women.

Data were collected through interview by trained medical students, held at the front door of participants’ houses. 

**Data** **analysis**

To score participants’ awareness about questions regarding general awareness about epilepsy, its causes, symptoms of seizures, and first-aid measures at the time of seizures, one point was awarded to each correct answer. Based on participants’ awareness score percentiles, their level of awareness about epilepsy was categorized as very low for 20^th^ percentile or lower; low 21^st^-40^th^; moderate 41^st^-60^th^; high 61^st^-80^th^; and very high for more than 80^th^. 

To analyze participants’ attitudes, “I strongly agree” and “I agree” were considered as “I agree”; and “I strongly disagree” and “I disagree” were considered as “I disagree”. 

To analyze participants’ first-aid measures at time of the last witnessed seizure, their first-aid measures were categorized as helpful, or harmful. One point was awarded to taking each helpful measure, or not taking each harmful measure. Based on participants’ first-aid measures score percentiles, their level of knowledge about epilepsy was categorized as low for 50^th^ percentile or lower; high 51^st^-75^th^; and very high for more than 75^th^. 

For the key proportions using exact binominal distribution, the 95% confidence interval (95% CI) was reported. Categorical variables were analyzed by Chi-Square test. For analyzing the differences among means of two groups and three groups or more, independent-sample t-test and one-way analysis of variance (ANOVA) test were used, respectively. Statistical analyses were performed using IBM SPSS Statistics 21 (Chicago, IL, USA). A probability level of less than 0.05 was considered significant.

## Results

Overall, 883 individuals participated in the survey. Interview sessions of 50 individuals were left incomplete and responses of 833 were analyzed. The mean (SD) age of participants was 36.2 (13.3), range=18 to 80 yr. Other socio-demographic characteristics of participants are shown in Table 1. 

**Awareness**

The mean (SD) total awareness score of participants was 22.8(11.3), range=4 to 51. The level of awareness about 41 (4.9%) was very high, 194 (23.3%) high, 255 (30.6%) moderate, 210 (25.2%) low, and 133 (16.0%) very low. 

Participants’ awareness about epilepsy is presented in six categories including 1) general awareness, 2) causes, 3) symptoms of seizures, 4) seizure triggers, 5) first-aid measures, and 6) recommended treatments. 

**General** **awareness**

The mean (SD) score about general awareness about epilepsy was 4.6(3.0), range=0 to 11. Almost two-thirds of participants knew that epilepsy is a non-contagious neurological disorder. However, 438 (52.8%) of respondents did not know for how long PWE should take their medications, and only 95 (11.4%) reported they had to take them lifelong. The 11 questions about general awareness are presented in Table 2. 

**Awareness about** **causes of epilepsy**

The mean (SD) awareness score about causes of epilepsy was 5.8 (3.4), range=0 to 13. Almost one-third of participants, 260 (31.2%), named at least one superstitious cause for epilepsy. Although 506 (63.3%) reported that epilepsy is not caused by demonic possession or evil spirits, 234 (29.6%) named insanity as a cause of epilepsy. As many as 126 (15.7%) participants mentioned evil eye as a cause of epilepsy. Among these 126 individuals, 84 (66.7%) did not know or reported that epilepsy is not treatable (Table 3).

**Awareness** **about symptoms**

The mean (SD) awareness score about symptoms of seizures was 7.0 (4.0), range=0 to 13. More than three-fourths of respondents knew that foaming at the mouth and whole body shaking are symptoms of seizures; however, almost half of the respondents ignored falling as a symptom of seizures (Table 4).

**Awareness** **about seizure triggers**

In terms of seizure triggers, 576 (69.5%) of respondents knew that not taking anticonvulsants on a regular basis could trigger seizures. Furthermore, almost half of them did not know whether physical exertion could trigger seizures (Table 5). 

The proportion of participants who reported that not taking anticonvulsants on a regular basis could trigger seizures was higher among those who considered epilepsy to be a treatable disorder compared to those who did not: 308(80.4%; 95% CI: 76.1-84.6) versus 264(60.4%; 95% CI: 55.8-65.3), *P*<0.001.

**Awareness** **about first-aid measures**

The mean (SD) awareness score about first-aid measures at time of seizures was 7.5(3.4), range=0 to 14. Among respondents, 367 (45.0%) did not know if they are supposed to roll a seizing person carefully on one side. Participants’ awareness of helpful or harmful measures is presented in Table 6. As many as 93 (11.6%) reported they would draw a chalk outline around the seizing person. 

**Awareness about treatment**

About treatment of epilepsy, 809 (98.5%) participants stated that a person with epilepsy needs to go to a physician. However, some suggested other measures including making religious vows 217 (28.6%); making a sacrifice 184 (24.3%); herbal treatment 176 (23.4%); tying a Dakhil, which is a piece of fabric tied to a shrine in the hope of treatment 142 (18.8%); acupuncture 140 (18.6%); and referring to Prayer-Seller, paid to write prayers for healing 81 (10.7%).

**Analysis of participants’ total awareness score**

The mean (SD) awareness score of participants of Fars ethnicity was significantly higher compared to those of other ethnicities: 24.1(11.1); 95% CI: 23.1-25.1 versus 20.1(11.3); 95% CI: 19.7-22.2, *P*<0.001. 

The mean (SD) score of participants with high school diploma or higher degrees was significantly higher compared to those with lower education: 26.9(11.1); 95% CI: 25.7-28.0 versus 19.7(10.5); 95% CI: 18.7-20.6, *P*<0.001. 

The mean (SD) score of participants with management, professional or clerical occupations and students was significantly higher compared to those who worked as service or sales workers, craft and related trade workers, were unemployed, or housewife: 25.7(11.7); 95% CI: 24.1-27.3 versus 21.8(11.1); 95% CI: 20.9-22.8 *P*<0.001. 

Among participants, 274 (32.9%) knew a person with epilepsy. The mean (SD) score of participants who knew someone with epilepsy was significantly higher compared to those who did not: 26.3(10.3); 95% CI: 25.1-27.6 versus 21.1(11.5); 95% CI: 20.1-22.1, *P*<0.001. 

No significant differences were observed between participants’ awareness scores and sex or age.

**Self-** **evaluation** **about the level of** **information**

Only 65 (7.8%) participants felt that they had information about epilepsy, while 434 (52.1%) did not find their level of information sufficient, and 334 (40.1%) considered it to be to some extent sufficient. Consequently, 634 (76.1%) felt the need for further training in epilepsy.

**Sources** **of** **information**

Overall, 597 (71.7%) participants gained their information about epilepsy from audiovisual media, the leading source of information. As many as 279 (33.5%) gained their information from their family and relatives, 258 (30.9%) print media, 179 (21.5%) healthcare professionals, 54 (6.5%) teachers, 48 (5.7%) colleagues, 39 (4.7%) the Internet, and 28 (3.4%) pamphlets and posters. Participants could name more than one source of information.

**Attitudes**

Attitudes of participants towards PWE were generally positive, as more than 90% of respondents stated that epilepsy is nothing to hide or be ashamed of, and PWE should inform others of their condition. The majority of respondents, 91.8%, stated that PWE can cope with their everyday life, and they can live a normal life. However, 32.6% stated that PWE cannot have the same quality of life as others. More than four-fifth of respondents reported that PWE are as intelligent as others; however, almost 30% stated that they cannot reach high levels of education. In terms of carrier life of PWE, 72% would work with them, and 67% stated that epilepsy is not an obstacle to hire a qualified person. The most negative attitudes against PWE were related to their marriage and having kids (Table 7). 

**First** **-** **aid** **measures**

Of 833 participants, 419 (50.3%) had witnessed at least one seizure in real life, and 65 (7.8) participants had seen seizures in the media. Of those who had witnessed seizure in real life, 174 (41.5%) took some measures, 123 (29.4%) did nothing, and 122 (29.1%) did not know what to do. The proportion of participants who took first-aid measures at the last witnessed seizure was higher among those who found their level of information about epilepsy sufficient compared to those who found it insufficient: 124 (55.9%; 95% CI: 49.4-62.3) versus 44 (23.5%; 95% CI: 17.4-29.6), *P*<0.001. As many as 122 (70.5%) of participants had tried to prevent the seizing person from getting injured by falling or sharp objects. However, 99 (56.4%) did not roll them on their side, and 70 (40.7%) even tried to restrain them to stop the seizure. More details are presented in Table 8. As many as 72 (41.4%) stated they were scared when they witnessed a seizure. Twelve participants reported that they drew an outline around the seizing person which was neither helpful nor harmful.

The mean (SD) safe first-aid measures score of participants was 9.9(2.1), range=5 to 14. The level of first-aid measures score of 74(42.5%) participants was very high, 79(45.4%) high, 21(12.1%) low. 

The mean (SD) score of participants of Fars ethnicity was significantly higher compared to those of other ethnicities: 10.5(1.9); 95% CI: 10.1-10.8 versus 8.9(1.9); 95% CI: 8.5-9.5, *P*<0.001.

The mean (SD) score of participants with high school diploma or higher degrees was significantly higher compared to those with lower education: 10.4(1.9); 95% CI: 9.9-10.8 versus 9.1(2.1); 95% CI: 8.8-9.5, *P*<0.001.

The mean (SD) score of participants with management, professional or clerical occupations and students was significantly higher compared to those who worked as service or sales workers, craft and related trade workers, were unemployed, or housewife: 10.6(1.9); 95% CI: 10.0-11.2 versus 9.4(2.1); 95% CI: 9.0-9.7 *P*=0.001. 

The mean (SD) score of participants who knew someone with epilepsy was significantly higher compared to those who did not: 10.0(2.0); 95% CI: 9.6-10.3 versus 9.2(2.0); 95% CI: 8.8-9.6, *P*=0.008. 

No significant differences were observed between participants’ first-aid measures scores and their sex, age, marital status, number of children, and whether they were scared at time of seizure.

The mean (SD) total awareness score of participants who had witnessed a seizure was significantly higher compared to those who had not: 24.8(10.9); 95% CI: 23.9-25.8 versus 20.2(11.2); 95% CI: 19.0-21.4, *P*<0.001. 

## Discussion

**Awareness**

The mean (SD) total awareness score of participants was 22.8(11.3), the level of awareness of 4.9% of participants was very high, 23.3% high, 30.6% moderate, 25.2% low, and 16.0% very low, which is altogether considered as insufficient. The mean total awareness score of participants who knew someone with epilepsy was 24% higher compared to those who did not. In addition, participants who had witnessed seizure had better awareness scores compared to those who had not. This may be suggestive of an association between previous encounter with PWE and gaining information about the disease.

People gained their information about epilepsy mostly from audiovisual media, which highlights the opportunity for policymakers to benefit from healthcare professionals and employ mass-media to educate the community accurately and avoid misbeliefs. The key role of healthcare professionals in providing the PWE, their families, and the society with proper information about health conditions has been emphasized in other studies as well (8, 22, 23). Some 11% of participants thought that demonic possession or evil spirits could give rise to epilepsy, which is less than the 20.3% in another study (24). Some 16% considered evil eye to be a cause for epilepsy. In Jordan, 28% of university students mentioned evil eye as a cause for epilepsy (21). Regarding recommended treatments for epilepsy, 98.5% reported that a person with epilepsy needs to refer to a physician. However, only 35.7% of respondents in a study reported that physicians should be the ones to treat epilepsy (25). Participants who knew that epilepsy is treatable were more likely to believe that not taking anticonvulsants regularly could trigger seizures. This could highlight the association between proper awareness about epilepsy and following the treatment properly. 

**Table 1:** Socio-demographic characteristics of participants

Variable	N (%)
Sex	
Male	467(56.1)
Female	366(43.9)
Marital status	
Married	528(63.4)
Never married	237(28.5)
Widowed	43(5.1)
Divorced	25(3.0)
Ethnicity	
Fars	511(61.4)
Azari	189(22.7)
Kord	37(4.4)
Lor	37(4.4)
Other	59(7.1)
Literacy	
Illiterate	26(3.2)
Primary school	58(7.3)
Middle school	108(13.5)
High school and diploma	254(31.8)
Associate degree	83(10.4)
Bachelor	176(22.1)
Master and PhD	93(11.7)
Occupation	
Managers	17(2.2)
Professionals	78(10.1)
Technicians	45(5.7)
Clerical supports	126(16.3)
Services and sales	106(13.6)
Craft workers	72(9.3)
Machine operators	30(3.8)
Other	20(2.6)
Unemployed	35(4.5)
Student	89(11.5)
Housewife	159(20.4)

**Table 2:** General awareness about epilepsy

Statement	It is true (%)	It is false (%)	I don’t know (%)
Epilepsy is a neurological disorder	569(68.9)	37(4.5)	220(26.6)
Most seizures are controlled after regular drug therapy	476(57.3)	43(5.2)	311(37.5)
Epilepsy is treatable	385(46.7)	152(18.5)	287(34.8)
There are different types of epilepsy	363(44.9)	50(6.2)	395(48.9)
Seizures might be transient and not be sensed by others	312(37.6)	104(12.6)	413(49.8)
PWE need to take lifelong medications	297(35.8)	95(11.4)	438(52.8)
Some PWE may sense seizure shortly before it happens	297(36.6)	87(10.7)	428(52.7)
Epilepsy is a psychological disorder	242(30.3)	314(39.3)	243(30.4)
Once being seizure free, drugs can be withdrawn immediately	107(12.9)	319(38.4)	404(48.7)
A normal EEG rules out epilepsy	97(11.6)	222(26.7)	513(61.7)
Epilepsy is a contagious disease	72(8.8)	522(64.1)	221(27.1)

**Table 3:** Awareness about etiology of epilepsy

Cause of epilepsy	Yes (%)	No (%)	Don’t know (%)
Real Causes			
Traumatic brain injury	464(57.3)	125(15.4)	221(27.3)
Genetic influence	422(52.6)	116(14.4)	265(33.0)
Birth injuries	424(52.1)	130(16.0)	259(31.9)
Brain tumor	350(43.5)	157(19.5)	297(37.0)
Drug side effects	298(37.4)	187(23.5)	311(39.1)
Unknown	187(23.5)	228(28.7)	380(47.8)
Prenatal conditions	117(14.8)	245(30.9)	431(54.3)
Stroke	196(24.4)	218(27.3)	386(48.3)
Superstitious causes			
Insanity	234(29.6)	241(30.5)	315(39.9)
Evil eye	126(15.7)	472(59.0)	202(25.3)
Curse	108(13.5)	494(61.9)	196(24.6)
Witchcraft	91(11.4)	513(64.4)	192(24.2)
Demonic possession	90(11.3)	506(63.3)	203(25.4)

**Table 4:** Awareness about possible symptoms of seizures

Events at the onset of seizures	Yes (%)	No (%)	Don’t know (%)
Whole body shaking	631(77.1)	37(4.5)	151(18.4)
Foaming at the mouth	622(77.0)	49(6.1)	136(16.9)
Loss of consciousness	520(64.2)	83(10.3)	206(25.5)
Tongue biting	518(65.1)	66(8.3)	212(26.6)
Body stiffening	489(61.2)	63(7.9)	247(30.9)
Tremor	467(58.1)	105(13.1)	231(28.8)
Body jerks	450(55.8)	101(12.5)	256(31.7)
Falling	410(51.1)	115(14.3)	278(34.6)
Confusion	399(50.7)	93(11.8)	295(37.5)
Head drop	371(46.8)	101(12.8)	320(40.4)
Staring	323(41.4)	144(18.4)	314(40.2)
Postictal state	281(35.5)	144(18.2)	367(46.3)
Urinary incontinence	250(31.9)	173(22.1)	361(46.0)

**Table 5:** Awareness about seizure triggers

Seizure triggers	Yes (%)	No (%)	Don’t know (%)
Taking drugs irregularly	576(69.5)	28(3.4)	224(27.1)
Anger	514(62.8)	34(4.2)	270(33.0)
Anxiety	507(61.7)	52(6.3)	263(32.0)
Sleep deprivation	387(47.4)	53(6.5)	376(46.1)
Alcohol consumption	383(46.8)	72(8.8)	363(44.4)
Drug abuse	340(42.3)	88(11.0)	375(46.7)
Physical exertion	287(35.3)	123(15.1)	404(49.6)
Smoking	236(29.0)	144(17.6)	435(53.4)
Studying too much	143(17.6)	228(28.0)	442(54.4)

**Table 6:** Awareness about first-aid measures at time of seizures

Measures	Yes (%)	No (%)	Don’t know (%)
Helpful measures			
Staying with them until ambulance arrives	713(87.0)	26 (3.20)	80 (9.80)
Clearing the area of dangerous objects	680(83.0)	36 (4.40)	103(12.6)
Preventing them from falling	675(82.7)	25 (3.10)	116(14.2)
Loosening clothing around the person’s	620(76.1)	44 (5.40)	151(18.5)
neck			
Taking them to hospital right after seizures	449(55.8)	178(22.1)	178(22.1)
Putting a pillow under their neck	428(52.5)	120(14.7)	268(32.8)
Rolling them carefully on their side	356(43.7)	92(11.30)	367(45.0)
Potentially harmful measures			
Attempting to open the mouth to put something between jaws	542(66.5)	88(10.80)	185(22.7)
Trying to restrain the person	308(38.0)	255(31.5)	247(30.5)
Shouting and moving to wake them up	287(35.4)	316(39.0)	208(25.6)
Sprinkling water on face to awaken them	284(35.1)	282(34.8)	244(30.1)
Administering drugs orally	180(22.2)	372(45.9)	258(31.9)
Pouring water with sugar into their mouth	134(16.5)	419(51.6)	259(31.9)
Forcing water down their throat	107(13.1)	457(56.4)	245(30.5)

**Table 7:** Attitudes of participants towards epilepsy

Statements about attitudes towards epilepsy	N	Agree (%)	Disagree (%)
PWE should inform partners before marriage	720	685(95.1)	35(4.9)
PWE should inform employers of their disease	672	630(93.7)	42(6.3)
Epilepsy is not a health condition to be ashamed of	607	561(92.4)	46(7.6)
I would not be embarrassed if someone in family had epilepsy	609	545(89.5)	64(10.5)
PWE should not hide their condition	605	534(88.3)	71(11.7)
PWE can cope with everyday life	584	533(91.3)	51(8.7)
PWE can have a normal life	570	523(91.8)	47(8.2)
PWE can be as successful in carriers as others	595	514(86.4)	81(13.6)
I would let my child play with a kid with epilepsy	618	499(80.7)	119(19.3)
Epilepsy is not a type of insanity	535	420(78.5)	115(21.5)
I would work with a person with epilepsy	567	412(72.7)	155(27.3)
PWE are as intelligent as others	503	413(82.1)	90(17.9)
PWE can achieve high levels of education	511	367(71.8)	144(28.2)
As an employer, I would hire a person with epilepsy	520	344(66.2)	176(33.8)
I am not afraid of being alone with a person with epilepsy	579	326(56.3)	253(43.7)
PWE have the same life quality as others	488	329(67.4)	159(32.6)
PWE can have kids	457	212(46.4)	245(53.6)
I would allow my child to marry a person with epilepsy	581	192(33.0)	389(67.0)
The society doesn’t discriminates against PWE	451	148(32.8)	303(67.2)
I would marry a person with epilepsy	492	108(22.0)	384(78.0)

**Table 8:** First-aid measures of participants at time of the last witnessed seizure

Measures	n (%)
Helpful measures	
Staying with the seizing person until medical personnel arrive	131(76.2)
Preventing them from falling	122(70.5)
Clearing the area of dangerous or sharp objects	119(68.8)
Loosening tight clothing around the person’s neck	107(61.8)
Putting a pillow under their neck	80(46.2)
Rolling them carefully on their side	75(43.6)
Taking them to the hospital right after seizures	30(17.2)
Harmful measures	
Attempting to open the mouth to put something between jaws	106(61.3)
Trying to restrain the person	70(40.7)
Sprinkling water on their face to wake them up	49(28.3)
Shouting and moving them in attempt to wake them up	45(26.2)
Forcing water down their throat	15(8.8)
Administering drugs orally	14(8.1)
Pouring water with sugar into their mouth	14(8.2)

**Attitudes**

Attitudes of participants towards PWE were generally positive. Respondents expressed the most positive attitudes towards PWE in not being ashamed of the disease and not hiding epilepsy. Some 67% reported epilepsy is not an obstacle to hire a qualified person; however, in a study, only 30.2% of respondents reported they would not discriminate against PWE as an employer (26). In communities where there are more negative attitudes towards epilepsy, PWE have a lower rate of employment (27-29).

Respondents expressed negative attitudes towards PWE in their marriage and having kids. Negative attitudes towards marriage of PWE have been reported in Nigeria and United Arab Emirates as well (16, 30). This could indicated that attitudes are positive as far as they do not interfere with personal interests. It still remains to be answered to what extent could the attitudes expressed in the studies reflect attitudes in real life (31).

**First-aid measures**

The mean (SD) safe first-aid measures score of participants at the last witnessed seizure was 9.9(2.1), the level of first-aid measures score of 42.5% participants was very high, 45.4% high, and 12.1% low, which is altogether considered as fairly helpful. Participants with epilepsy or had witnessed a seizure had taken better first-aid measures at the last witnessed seizure. Previous encounter with epilepsy sensitizes one to gain information, which could highlight the positive influence of proper knowledge over first-aid measures. The positive influence of proper awareness over first-aid measures has been emphasized in some other studies as well (25, 32-34). Participants who did not consider their level of information about epilepsy to be sufficient were more likely to be doubtful and not take any first-aid measures at the last witnessed seizure. Confidence about proper awareness about epilepsy is a predictor of taking first-aid measures while witnessing a seizure. Therefore, it is essential to design epilepsy-related education programs in a way to increase people’s confidence about their level of information about epilepsy. Some 70% of participants tried to prevent the seizing person from falling or being injured. However, less than half of participants rolled the seizing person on one side, which could have been life-threatening. In addition, less than one-fifth reported they had taken the patient to the hospital. People in Iran do not usually call an ambulance unless in cases of trauma or heart attack (22).

The audiovisual media be used as means of unconscious training considering their potentials, especially. It might be more effective to include a documentary or a role-playing of a seizing person in epilepsy-related training programs. Informative training programs could enhance one’s confidence in taking the right first-aid measures while encountering a seizing person.

**In conclusion **the awareness of people of Tehran about epilepsy was insufficient, attitudes were generally positive but rather conservative, and first-aid measures at the last witnessed seizure were fairly helpful. Previous encounter with epilepsy positively influenced taking first-aid measure at time of the last witnessed seizure.

We recommend that the audiovisual media be used as means of unconscious training considering their potentials, especially. It might be more effective to include a documentary, or a role playing of a seizing person in epilepsy-related training programs. Informative training programs could enhance one’s confidence in taking the right first-aid measures while encountering a seizing person.

## References

[1] Noble AJ, Marson AG (2016). Should we stop saying "epileptic"? A comparison of the effect of the terms "epileptic" and "person with epilepsy". Epilepsy Behav.

[2] Mula M, Sander JW (2016). Psychosocial aspects of epilepsy: a wider approach. BJ Psych Open.

[3] Alkhamees HA, Selai CE, Shorvon SD (2015). The beliefs among patients with epilepsy in Saudi Arabia about the causes and treatment of epilepsy and other aspects. Epilepsy Behav.

[4] Preston SM, Shihab N, Volk HA (2013). Public perception of epilepsy in dogs is more favorable than in humans. Epilepsy Behav.

[5] Hounsossou CH, Queneuille JP, Ibinga E, Preux PM, Dalmay F, Druet-Cabanac M (2015). Knowledge, attitudes, and behavior among key people involved in the employment of people with epilepsy in southern Benin. Epilepsy Behav.

[6] Aydemir N (2011). Familiarity with, knowledge of, and attitudes toward epilepsy in Turkey. Epilepsy Behav.

[7] Eze CN, Ebuehi OM, Brigo F, Otte WM, Igwe SC (2015). Effect of health education on trainee teachers' knowledge, attitudes, and first aid management of epilepsy: An interventional study. Seizure.

[8] O'Hara KA (2007). First aid for seizures: the importance of education and appropriate response. J Child Neurol.

[9] Lim KS, Wu C, Choo WY, Tan CT (2012). Development and validation of a public attitudes toward epilepsy (PATE) scale. Epilepsy Behav.

[10] Mecarelli O, Capovilla G, Romeo A, Rubboli G, Tinuper P, Beghi E (2010). Past and present public knowledge and attitudes toward epilepsy in Italy. Epilepsy Behav.

[11] Bagić A, Bagić D, Zivković I (2009). First population study of the general public awareness and perception of epilepsy in Croatia. Epilepsy Behav.

[12] Baker GA, Hargis E, Hsih MM (2008). Perceived impact of epilepsy in teenagers and young adults: an international survey. Epilepsy Behav.

[13] Elliott J, Shneker B (2008). Patient, caregiver, and health care practitioner knowledge of, beliefs about, and attitudes toward epilepsy. Epilepsy Behav.

[14] Frank-Briggs AI, Alikor EA (2011). Knowledge and attitudes of parents toward children with epilepsy. Ann Afr Med.

[15] Wu KN, Lieber E, Siddarth P, Smith K, Sankar R, Caplan R (2008). Dealing with epilepsy: parents speak up. Epilepsy Behav.

[16] Ekenze OS, Ndukuba AC (2013). Perception of epilepsy among public workers: perspectives from a developing country. Epilepsy Behav.

[17] El Sharkawy G, Newton C, Hartley S (2006). Attitudes and practices of families and health care personnel toward children with epilepsy in Kilifi, Kenya. Epilepsy Behav.

[18] Lim KS, Hills MD, Choo WY, Wong MH, Wu C, Tan CT (2013). Attitudes toward epilepsy among the primary and secondary school teachers in Malaysia, using the public attitudes toward epilepsy (PATE) scale. Epilepsy Res.

[19] Homi Bhesania N, Rehman A, Saleh Savul I, Zehra N (2014). Knowledge, attitude and practices of school teachers towards epileptic school children in Karachi, Pakistan. Pak J Med Sci.

[20] Y Young GB, Derry P, Hutchinson I, John V, Matijevic S, Parrent L (2002). An epilepsy questionnaire study of knowledge and attitudes in Canadian college students. Epilepsia.

[21] Hijazeen JK, Abu-Helalah MA, Alshraideh HA, Alrawashdeh OS, Hawa FN, Dalbah TA (2014). Knowledge, attitudes, and beliefs about epilepsy and their predictors among university students in Jordan. Epilepsy Behav.

[22] Kolahi AA, Abbasi-Kangevari M, Bakhshaei P, Mahvelati-Shamsabadi F, Tonekaboni SH, Farsar AR (2017). Knowledge, attitudes, and practices among mothers of children with epilepsy: A study in a teaching hospital. Epilepsy Behav.

[23] Kolahi AA, Tahmooreszadeh S (2009). First febrile convulsions: inquiry about the knowledge, attitudes and concerns of the patients' mothers. Eur Pediatr.

[24] Deresse B, Shaweno D (2016). General public knowledge, attitudes, and practices towards persons with epilepsy in South Ethiopia: A comparative community-based cross-sectional study. Epilepsy Behav.

[25] Ezeala-Adikaibe BA, Achor JU, Onwukwe J, Ekenze OS, Onwuekwe IO, Chukwu O (2013). Knowledge, attitude and practice towards epilepsy among secondary school students in Enugu, southeast Nigeria. Seizure.

[26] Bain LE, Awah PK, Takougang I, Sigal Y, Ajime TT (2013). Public awareness, knowledge and practice relating to epilepsy amongst adult residents in rural Cameroon—case study of the Fundong health district. Pan Afr Med J.

[27] Lee SA (2005). What we confront with employment of people with epilepsy in Korea. Epilepsia.

[28] Varma NP, Sylaja PN, George L, Sankara Sarma P, Radhakrishnan K (2007). Employment concerns of people with epilepsy in Kerala, south India. Epilepsy Behav.

[29] Clarke BM, Upton AR, Castellanos C (2006). Work beliefs and work status in epilepsy. Epilepsy Behav.

[30] Abduelkarem AR (2016). Societal problems that patients with epilepsy are facing in Sharjah, UAE. Epilepsy Behav.

[31] Novotná I, Rektor I (2017). The long-term development of public attitudes towards people with epilepsy in the Czech Republic: 1981, 1984, 1998 and 2009 studies. Acta Neurol Scand.

[32] Kaddumukasa M, Kakooza A, Kayima J, Kaddumukasa MN, Ddumba E, Mugenyi L (2016). Community knowledge of and attitudes toward epilepsy in rural and urban Mukono district, Uganda: A cross-sectional study. Epilepsy Behav.

[33] Gebrewold MA, Enquselassie F, Teklehaimanot R, Gugssa SA (2016). Ethiopian teachers: their knowledge, attitude and practice towards epilepsy. BMC Neurol.

[34] Kolahi AA, Ghorbanpur-Valukolaei M, Abbasi-Kangevari M, Farsar AR (2018). Knowledge, attitudes, and first-aid measures about epilepsy among primary school teachers in northern Iran. Acta Neurol Scand.

